# Scientific Items

**Published:** 1888-08

**Authors:** 


### SCIENTIFIC ITEMS.

**Long Distance Telegraphy.**—The recently announced cl im of a telegraphic circuit of over 6,000 miles, surpassing all previous experiments, is somewhat misleading. Many efforts at long circuit work have occurred during the past few years, the distance varying from 4,600 to 8,100 mil s.

It is a matter of considerable pride to the old operators of the Western Union Telegraph Company in San Francisco, says the San Francisco *Alta*, that the feat of transmitting clock signals through 7,200 miles line and communicating directly through that same line has never been equaled. The occasion of this feat was the telegraphic determination of the difference of longitude in time between the United States coast survey station in San Francisco and the observatory of the Harvard University at Cambridge, in the year 1869. In order to determine the time of transmission of a signal either from the clock or from the operator's key over the given length of the line of 3,600 miles, three different methods were devised. One of these was original with Prof. George Davidson, who had charge of the observations. Through the liberality of the management of the Western Union Telegraph Company, a double circuit of line was looped at Cambridge, so that there extended from the San Francisco observatory 3,600 miles to Cambridge, and the return from Cambridge by a somewhat different route of nearly equal length. The two "earths" were under the San Francisco observatory, distant from each other not more than ten feet. The line was first opened by an operator in the observatory, and when the fast connection was made at Cambridge, the San Francisco operator was considerably astonished to get his own message back within one second of time.

Then the astronomical break circuit clock was thrown into line, and made its first break on a pen recording upon a revolving cylinder of paper in the San Francisco observatory, and after this break had traversed the line to Cambridge, it returned and made a break upon a second pen moving parallel with the former, in about eight-tenths of a second of time. This was continued every second for several minutes, and was repeated upon several nights, and when one of the twelve batteries in this long circuit was removed, the wave length time was reduced to only sixty-five hundredths of a second. Communication was, of course, carried on at the same rate of speed. This feat over a line 7,200 miles in length has been unrivaled up to the present time, both as a practical working exhibit and a scientific success.—*Scientific American.*

**A Gigantic Oak.**—One of the sights at Havre Maritime Exhibition is the trunk of a gigantic oak placed in an iron boat especially constructed for its reception. This trunk was found accidentally in the bed of the Rhone, at La Balme, as long ago as 1874, when during a period of low water, a branch was observed projecting above the surface. On a closer examination, this was found to belong to a huge trunk embedded in the mud of the river. It was not till ten years later (1883) that the level of the water was low enough to enable the tree to be taken out. It took five months to remove it from the bed of the river, some 30 feet of sand and gravel having to be removed in order to liberate it. At length, on March 25, 1884, it was brought to the shore, where the dimensions of the trunk were found to be as follows: Length, 101.6 feet; circumference at the origin of the roots 29.5 feet; circumference at the level of the soil, 19.6 feet. The actual weight of the tree is 121,000 pounds, and its age is estimated to be from 400 to 450 years. The boat, called the Dryophore ("oak bearer"), is intended to transport the tree from river to river, for exhibition.—*Scientific American.*

**Simple Method of Artificial Respiration.**—In the *British Medical Journal* (*London Medical Record*), Mr. J. A. Francis describes a simple method of artificial respiration, which, he alleges, combines all the advantages of the Marshall Hall, Sylvester, and Howard methods, without any of their disadvantages. The plan is as follows: the body of the patient is laid on the back, with clothes loosened, and the mouth and nose wiped. Two bystanders pass their right hands under the body at the level of the waist, and grasp each other's hands, then raise the body until the tips of the fingers and the toes of the subject alone touch the ground; count fifteen rapidly; then lower the body flat to the ground, and press the elbows to the sides hard; count fifteen again; then raise the body again for the same length of time; and so on, alternately raising and lowering. The head, arms, and legs are to be allowed to dangle down quite freely when the body is raised. The author alleges that this method is most successful, and it is so simple that any one can perform it without any teaching.

**The** *Ledger*, May 29, 1888, says that M. Oscar Schmidt, professor at the University of Gratz, in Styria, has discovered a method by which pieces of living sponge can be broken off and planted in a favorable spot. From very small cuttings of this kind Professor Schmidt has obtained large sponges in the course of three years, at a very small expense. One of his experiments showed that the cultivation of 4,000 sponges had not cost more than 225 francs, including the interest for three years on the capital expended. The Austro Hungarian Government has been so much struck with the importance of these experiments that it has officially authorized the protection of this new industry on the coast of Dalmatia.—*Medical and Surgical Reporter.*

**In Oakland, Cal.**, and other places, compressed air is now successfully used for operating switches having an interlocking apparatus. The system is, in fact, very extensively used on several of our principal railways. It takes up less space than mechanical locking machinery, and the labor of working it is very light. The ground connections can be buried out of the way, and can be led out from the tower in any way most convenient.

